# The Viruses of *Botrytis cinerea* and Beyond: Molecular Characterization of RNA Viruses and Retroplasmids

**DOI:** 10.3390/v17121527

**Published:** 2025-11-21

**Authors:** Huang Huang, Jiasen Cheng, Yanping Fu, Qing Cai, Yang Lin, Tao Chen, Bo Li, Xiao Yu, Xueqiong Xiao, Daohong Jiang, Jiatao Xie

**Affiliations:** 1 National Key Laboratory of Agricultural Microbiology, Huazhong Agricultural University, Wuhan 430070, China; h.h@webmail.hzau.edu.cn (H.H.);; 2 The Provincial Key Lab of Plant Pathology of Hubei Province, College of Plant Science and Technology, Huazhong Agricultural University, Wuhan 430070, China; 3 Hubei Hongshan Laboratory, Wuhan 430070, China

**Keywords:** *Botrytis cinerea*, mycovirus, partitivirus, RNA satellite, retroplasmid, virosphere, mobile genetic element

## Abstract

Over the past five years, research has progressively revealed a rich diversity of RNA viruses in *Botrytis cinerea*. In this study, we identified nine RNA viruses from the viromes of three *B. cinerea* strains, including five mitoviruses, one umbra-like virus, and three partitiviruses. Among these, Sclerotinia sclerotiorum partitivirus 1 (SsPV1) was artificially introduced in a previous study. Excluding SsPV1, we cloned the other two partitiviruses and confirmed that both belong to *Gammapartitivirus* and contain three genomic segments, with dsRNA3 as an RNA satellite. In addition to RNA viruses, we discovered 12 retroplasmids in the three *B. cinerea* strains. These retroplasmids utilize the mitochondrial genetic codes and only encode a single open reading frame, which is predicted to produce a reverse transcriptase. It is also well known that mitoviruses use the mitochondrial genetic codes to encode their RNA-dependent RNA polymerase. Given the similarities between mitoviruses and retroplasmids in several aspects, we suggest that the mycovirus community could consider whether retroplasmids should be included within the conceptual scope of viruses. Furthermore, this study calls on researchers to pay attention to mobile genetic elements beyond typical RNA viruses, such as the retroplasmids reported here. Additionally, it underscores the importance of using single-spore or single-protoplast isolation methods in mycoviral studies to maintain a consistent genetic and viral background when investigating viral effects on the fungal host.

## 1. Introduction

*Botrytis cinerea* is a notable necrotrophic plant pathogenic fungus with an exceptionally broad host range, capable of infecting over 1600 plant species [1,2]. This fungus not only causes damage during crop growth but also continues to infect during the postharvest stage, leading to the reduced quality and yield of agricultural products. It is estimated that global annual expenditures for controlling gray mold exceed EUR 1 billion, with economic losses ranging between USD 10–100 billion [3,4]. Under specific climatic conditions, *B. cinerea* can develop a “noble rot” on grapes instead of common gray mold. Grapes with this unique infection are used to produce distinctively flavored botrytized wines, providing the fungus special value in the winemaking industry [5,6]. Given the importance of *B. cinerea* in agricultural production, studies on this pathogen hold significant practical and theoretical value.

Research on mycoviruses (also known as fungal viruses) has revealed remarkable viral diversity and evolutionary insights, and highlighted their potential for biocontrol in plant disease management [7,8]. The study of viruses infecting *Botrytis* spp. began with the detection of dsRNA and virus-like particles in *B. cinerea* [9,10,11]. Characterization of Botrytis virus F [12], Botrytis virus X [13], Botrytis cinerea negative-stranded RNA virus 1 [14], and Botrytis ourmia-like virus [15] revealed the evolutionary relationships among mycovirus and plant viruses. In recent decades, the application of high-throughput sequencing has led to the identification of over one hundred viruses in *B. cinerea*, underscoring both the high incidence of viral infections and common occurrence of mixed viral infections [16,17,18,19,20]. For example, integrated RNA-seq and RT-PCR analyses detected mycoviruses in approximately 100% of *B. cinerea* isolates from Italy, Spain, and Israel [17,20]. Duan et al. further confirmed complex virus co-infections and explored possible rules governing these co-infection patterns [20]. Current profiling of the *Botrytis* virome shows that it is dominated by dsRNA viruses, positive-sense single-stranded RNA viruses, and negative-sense single-stranded RNA viruses [21]. Thus far, only one species of circular single-stranded DNA virus has been reported in *B. cinerea*, which appears to be globally distributed [22,23,24]. In addition, numerous hypovirulence-associated viruses have been reported in *Botrytis* spp., including a mitovirus [25,26,27], a botybirnavirus [28], a fusagravirus [29], and a partitivirus [30], among others [31,32]. However, fully evaluating the biocontrol potential of these viruses requires further investigation into whether the hypovirulent strains harbor multiple viruses—a particular concern given the prevalence of mixed infections in *B. cinerea*—and whether the hypovirulence trait is consistently maintained across different *B. cinerea* isolates [10,11,21]. A feasible approach to address this concern is the construction of infectious clones—as has been successfully achieved with viruses such as Cryphonectria hypovirus 1 and Botrytis virus F in *B. cinerea* [33,34]. Given these developments, *B. cinerea* has been proposed as a model system for further advancing mycovirus studies [21].

RNA viruses are the most extensively described mobile genetic elements (MGEs) in fungi, largely due to the rise in viromic screens and their recognized biological significance. Recently, Koonin et al. categorized the virosphere within the replicator space into the orthovirosphere and perivirosphere, which are separated by fuzzy boundaries [35]. All recognized virus taxa in the International Committee on Taxonomy of Viruses (ICTV) belong to the orthovirosphere. Among them, certain capsidless RNA viruses in fungi, such as mitoviruses and narnaviruses, are situated near the boundary between the orthovirosphere and perivirosphere [35]. Elements such as viroids, viroid-like RNAs, group II introns, and non-long terminal repeat (non-LTR) retrotransposons reside within the perivirosphere [35]. Retroplasmids are fungal mitochondrial MGEs with circular or linear DNA genomes that encode only a reverse transcriptase [36]. Although linear retroplasmids share certain terminal genomic features with telomeres, phylogenetic analyses indicate that retroplasmids are more closely related to group II introns found in fungal mitochondria or bacteria [37,38]. Unlike group II introns, which are MGEs typically integrated into the host genome, retroplasmids are rarely integrated into the host mitochondrial genome during their replication cycle—though integration has been observed in some cases [39,40]. In addition, horizontal gene transfer from retroplasmids to the mitochondrial genome has been observed frequently compared to transfers from the mitochondrial genome to retroplasmids [41,42,43,44,45,46]. Generally, retroplasmids utilize the host mitochondrial RNA polymerase to transcribe their single gene, which encodes a reverse transcriptase (RT). The resulting RT then reverse transcribes the mRNA back into DNA to complete the replication cycle [36]. Since retroplasmids were found to exist as DNA and do not encode a coat protein, they were historically designated as plasmids—a naming convention similar to that used for fungal viruses, which had been referred to as RNA plasmids due to their lack of an extracellular phase, their lack of infectivity, and even the absence of a coat protein in some cases [47,48,49]. Furthermore, recently, a study revealed that the dynamics of some persistent fungal RNA viruses are similar to those of plasmids, highlighting a different lifestyle of mycoviruses [50]. Both retroplasmids and mitoviruses are capsidless elements residing in mitochondria that are generally not integrated into the mitochondrial genome [36,51]. However, although mitoviruses show clear phylogenetic relationships with known RNA viruses, retroplasmids are only distantly related to known fungal reverse-transcribing viruses within the LTR retrotransposon group [52]. These reverse-transcribing viruses possess LTRs at their genome termini, which are essential for integrating into the host genome. In addition, they encode a Gag polyprotein containing a capsid protein (CP) domain and a Pol polyprotein containing an RT domain [53,54]. Thus, it remains uncertain whether retroplasmids should be classified as viruses (i.e., within the orthovirosphere). Nonetheless, like group II introns and non-LTR retrotransposons, they could at least be categorized into the perivirosphere within the virosphere’s replicator space [35,52]. The overall diversity of retroplasmids in fungi remains largely unknown [42]. Characterizing these retroplasmids will contribute to a more comprehensive understanding of the fungal virosphere.

In this study, we employed RNA-seq and RT-PCR to characterize complex RNA virus infections in two *B. cinerea* isolates from prior research [55]. Through cloning the full-length genomes of the RNA viruses, we identified additional RNA satellites associated with two previously reported gammapartitiviruses. Moreover, we discovered diverse retroplasmids—a group of mitochondrial MGEs within the perivirosphere—for the first time in *B. cinerea*. Our findings highlight a previously overlooked component of the fungal virosphere.

## 2. Materials and Methods

### 2.1. Fungal Material

The *B. cinerea* strain KY-1 was isolated by transferring mycelia (with typical gray mold symptom) from a diseased blueberry fruit onto a potato dextrose agar (PDA) plate supplemented with 100 µg/mL cefotaxime. The blueberry was obtained from a market at the University of Kentucky, USA [55]. KY-1V1 and KY-1V2 were obtained by transfecting KY-1 protoplasts with SsPV1 virions [55]. In this study, we speculated that the “KY-1” strain previously used for SsPV1 transfection was not a single isolate. The *B. cinerea* strains were identified using primers listed in Table S1. KY-1V1(SsPV1-free) is a strain derived from KY-1V1 that lost SsPV1 during subculturing. The term “KY-1 serial strains” was used to refer to KY-1, KY-1V1, KY-1V1(SsPV1-free) and KY-1V2. KY-1T1 was acquired by re-transfecting KY-1 protoplasts with SsPV1 virions. B05.10 is a *B. cinerea* strain with a well-annotated genome [56]. B05.10V was acquired by transfecting B05.10 protoplasts with SsPV1 virions. *B. cinerea* was generally cultured on PDA medium at 20–22 °C in darkness.

### 2.2. Total RNA Extraction, RNA Sequencing

Fresh mycelia were harvested after being cultured on cellophane-overlaid PDA plates and ground in liquid nitrogen using a mortar and pestle. Total RNA was extracted from the finely powdered mycelia using the NI-*Sclerotinia sclerotiorum* RNA Reagent (NewBioIndustry, Shachuan Bio-technology Co., Ltd., Tianjin, China) according to the manufacturer’s instructions. Equal masses of total RNA were pooled and sent to Shanghai Majorbio Biopharm Technology Co. Ltd. (Shanghai, China) for RNA-seq, employing an rRNA depletion method for lncRNA library construction. Finally, approximately 9 Gb of Illumina paired-end (150 bp × 2) clean data were obtained from the Illumina HiSeq platform.

### 2.3. Contig Assembly and Virus Identification

RNA-seq data were processed by removing adapters and low-quality reads using Trimmomatic (v0.36) [57], followed by transcript assembly with MEGAHIT (v1.2.9) [58] or rnaviralspades.py (v3.15.4) [59]. The resulting contigs were subjected to open reading frame (ORF) prediction and translation using TransDecoder.LongOrfs (v5.7.1) (key parameters: -m 100 -G Mitochondrial-Protozoan). The translated protein sequences were then searched against the NR database using diamond blastp [60]. During preliminary screening, sequences with annotation results containing the keyword “virus” were retained for further manual analysis, including the elimination of false positives and removal of potential host sequences misassembled at the ends of contigs by BLASTn (v2.16.0) alignment against the NT database. The candidate RNA viruses were confirmed using primers listed in Table S2.

To confirm the presence of other viruses (or viral fragments) or virus-like molecules (such as retroplasmids) in the sequencing data, the following three types of sequences were further analyzed: (1) Contigs with no ORFs predicted; (2) Contigs with ORFs that had no functional annotations; (3) Contigs whose best matches were not proteins from *B. cinerea* and exhibited less than 90% amino acid identity. For these sequences, additional identification methods were employed, including BLASTn (v2.16.0) search against the NT database, ORF prediction using different genetic codes, conserved domain analysis via MOTIF search (https://www.genome.jp/tools/motif/ (accessed on 18 May 2025)), and online HHpred search (https://toolkit.tuebingen.mpg.de/tools/hhpred (accessed on 18 May 2025)) [61]. The retroplasmids were further detected using primers listed in Table S3.

### 2.4. Virus Terminal Sequence Determination

The terminal sequences of viral RNAs were cloned following a previously described method [62]. Initially, attempts were made to obtain all viral RNA termini through RNA ligase-mediated rapid amplification of cDNA ends (RACE). In this approach, a 5′ phosphorylated PC3-T7 loop adapter was ligated to the 3′ ends of RNAs, followed by reverse transcription using M-MLV. The resulting cDNA was amplified by PCR with a PC2 primer and virus-specific primer. The PCR products were subsequently cloned into the T-vector pMD18-T for Sanger sequencing. For termini that failed to obtain via RNA ligase-mediated RACE, an alternative RACE strategy was utilized [62]. For 5′ RACE, a virus-specific primer located approximately 500–700 bp from the terminus was used to reverse transcribe the 5′ terminal region to cDNA. A poly(dG) or poly(dA) tail was then added to the 3′ end of the cDNA using terminal deoxynucleotidyl transferase. Amplification of the candidate 5′ terminal sequence was performed using a tail-adaptor-poly(dC) primer (or tail-adaptor-poly(dT) primer) together with a virus specific primer. For 3′ RACE, a poly(A) or poly(C) tail was first added to 3′ end of the RNA using poly(A) polymerase. The tailed RNA was reverse transcribed using a tail-adaptor-poly(dT) or tail-adaptor-poly(dG) primer. The terminal sequence was then amplified via PCR with the corresponding tail-adaptor and a virus-specific primer. A nested PCR step was incorporated when necessary to improve specificity or yield. Primers used for terminal cloning were listed in Table S4.

### 2.5. Phylogenetic Tree Construction and Visualization

Viral protein sequences were aligned using MUSCLE (v5.1.0) [63]. The resulting multiple sequence alignment was reordered with esl-alimanip. The alignment results were inspected using Jalview software (v2.11.4.1). Subsequently, the multiple sequence alignment was trimmed with ClipKIT (v2.1.3) [64]. Using the trimmed alignment, a phylogenetic tree was constructed with IQ-TREE (v2.3.6) [65]. The resulting phylogenetic tree was visualized using the R package ggtree (v3.16.3) [66], and final layout and esthetic refinements were completed with Adobe Illustrator (v2021).

## 3. Results

### 3.1. KY-1 and KY-1V1 Are Two Distinct Fungal Strains of B. cinerea

**Figure 1:**
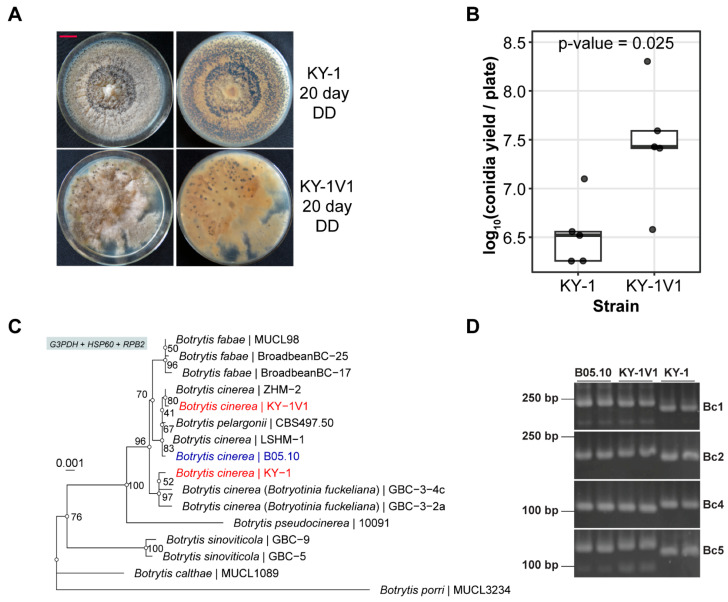
Species identification of the strains KY-1 and KY-1V in *Botrytis* spp. (**A**) Colony morphologies of KY-1 and KY-1V1. All strains were cultured under continuous dark (DD) conditions for 20 days. (**B**) Conidia yield of KY-1 and KY-1V1. (**C**) Phylogenetic analysis of the strains KY-1 and KY-1V1 based on the concatenated G3PDH, HSP60 and RPB2 genes. The nucleotide sequences of the three genes were aligned using Clustal Omega (v1.2.4). The alignments were trimmed manually to remove gaps at the ends and concatenated by catfasta2phyml (written by Johan A. A. Nylander). The phylogenetic tree was inferred by iqtree2 (v2.2.6) with the arguments “-B 1000 -m MFP + MERGE -rcluster 10”. *Botrytis porri* was set as the outgroup. The ultrafast bootstrap values were noted at the internal node. (**D**) Discrimination of KY-1 and KY-1V1 by amplifying the microsatellite loci of *B. cinerea*. The PCR products corresponding to the microsatellite loci with different sizes in 4% agarose electrophoresis were displayed.

Previously, we transferred SsPV1 to a *B. cinerea* strain KY-1, which was isolated from diseased blueberry, generating a series of transformants (e.g., KY-1V1 and KY-1V2). By comparing the colony morphologies of KY-1 and KY-1V1, it was found that KY-1 produced more sclerotia than KY-1V1, while KY-1V1 exhibited higher conidia production than KY-1 (Figure 1A,B) [55]. The strain KY-1 was preliminarily identified as *B. cinerea* solely through sequencing of the internal transcribed spacer (ITS) region followed by BLASTn [55]. In this study, we performed a phylogenetic analysis of partial regions of the glyceraldehyde-3-phosphate dehydrogenase (G3PDH) gene, heat shock protein 60 (HSP60) gene, and DNA-dependent RNA polymerase II subunit (RPB2) gene for strain KY-1 and KY-1V1 [67,68]. Phylogenetic analysis based on the concatenated sequences of three genes confirmed that both KY-1 and KY-1V1 belonged to *B. cinerea* (Figure 1C). Nevertheless, KY-1V1 showed a closer phylogenetic relationship to strain B05.10, whereas KY-1 was more closely related to *B. cinerea* strain GBC-3-4c. KY-1 and KY-1V1 were located on distinct branches within the *B. cinerea* species (Figure 1C). Additionally, using microsatellite marker primers developed by Fournier et al. for *B. cinerea* [69], the band sizes amplified using primers Bc1, Bc2, Bc4, and Bc5 were noticeably different from those of KY-1V1 and B05.10, while the differences between KY-1V1 and B05.10 were relatively minor (Figure 1D). Results from both methods consistently indicated that although KY-1 and KY-1V1 both belong to *B. cinerea*, they represent distinct strains, with KY-1V1 being more closely related to strain B05.10. The observed contradiction raised the possibility that the “KY-1” strain subjected to SsPV1 transfection was not a single, purified culture of *B. cinerea*, but rather a mixed culture of both the KY-1 and KY-1V1(SsPV1-free) strains [55].

### 3.2. KY-1 and KY-1V1 Contain Different Virus Composition

**Figure 2:**
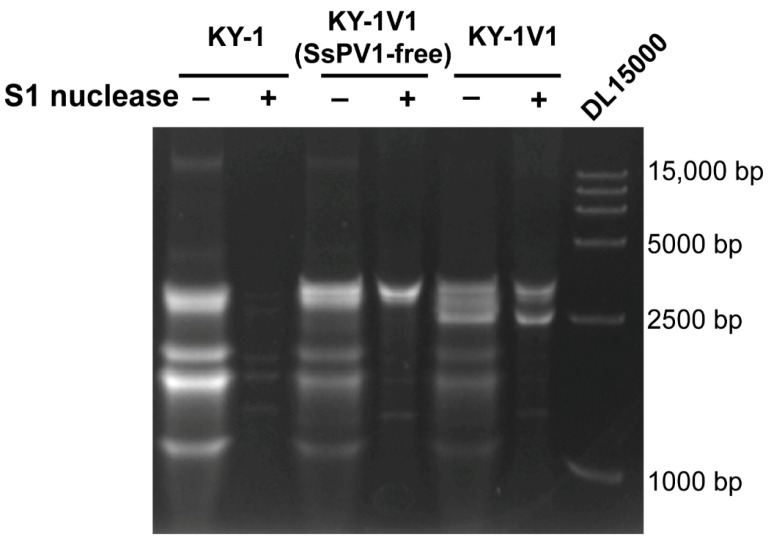
dsRNA profiles of KY-1, KY-1V1(SsPV1-free) (a strain cured of SsPV1), and KY-1V1. Single-stranded RNA was digested with S1 nuclease. A DNA ladder (DL15000) was included on the right for size reference.

We found that in the dsRNA of KY-1 and KY-1V1, in addition to SsPV1 band at approximately 2500 bp, other dsRNA bands were present. As shown in Figure 2, after the extracted dsRNA was treated with S1 nuclease to degrade ssRNA, bands larger than 2500 bp were observed in both KY-1V1 and the SsPV1-free strain KY-1V1(SsPV1-free). Moreover, three bands ranging in size from 1000 bp to 2500 bp were detected in KY-1, KY-1V1(SsPV1-free), and KY-1V1. These results indicated the presence of other RNA viruses in the KY-1 and KY-1V1 strains.

**Figure 3:**
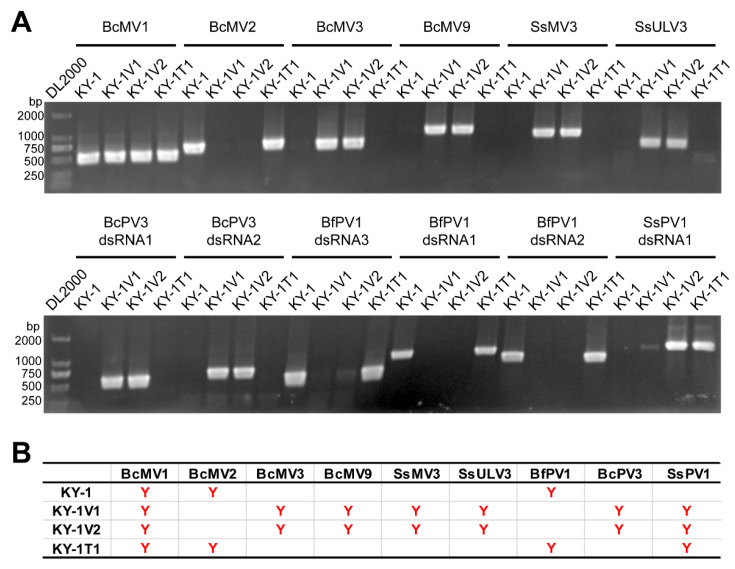
RNA viruses in *B. cinerea* strains KY-1 and KY-1V1. (**A**) Detection of viruses in KY-1 and KY-1V1 by RT-PCR. Both KY-1V1 and KY-1V2 are strains previously obtained carrying SsPV1. KY-1T1 was obtained by re-transfecting SsPV1 into KY-1. (**B**) Summary table of RNA virus detection results in the KY-1 series strains. The letter “Y” indicates the presence of the virus in the strain.

To characterize these unknown putative dsRNA viruses, total RNA from the KY-1 serial strains and the well-annotated reference strain B05.10 were subjected to rRNA-depleted RNA sequencing. Following validation of the candidate viral contigs by RT-PCR, the RNA viruses of KY-1, KY-1V1 and KY-1V2 were confirmed (Figure 3). Strain KY-1 harbored three viruses: Botrytis cinerea mitovirus 1 (BcMV1), Botrytis cinerea mitovirus 2 (BcMV2), and Botryotinia fuckeliana partitivirus 1 (BfPV1). Strain KY-1V1 contained seven viruses: SsPV1, BcMV1, Botrytis cinerea mitovirus 3 (BcMV3), Botrytis cinerea mitovirus 9 (BcMV9), Sclerotinia sclerotiorum mitovirus 3 (SsMV3), Sclerotinia sclerotiorum umbra-like virus 3 (SsULV3), and Botrytis cinerea partitivirus 3 (BcPV3). Strain KY-1V2 shared same RNA viruses with KY-1V1 (Figure 3).

**Figure 4:**
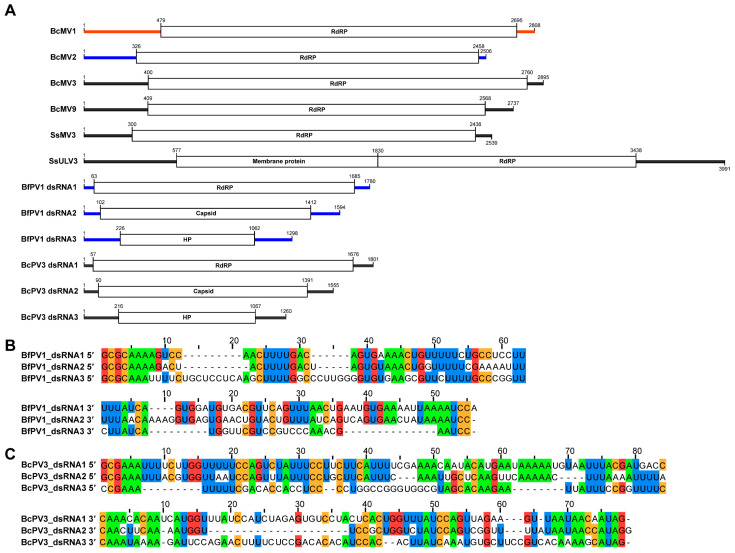
RNA viruses newly identified in KY-1 and KY-1V1. (**A**) Genomic organization. HP denotes hypothetical protein, and RdRP denotes RNA-dependent RNA polymerase. The red line indicates BcMV1, which is common to both KY-1 and KY-1V1. The blue and black lines represent viruses unique to KY-1 and KY-1V1, respectively. (**B**) Sequence conservation of 5′ and 3′ terminal of BfPV1. (**C**) Sequence conservation of 5′ and 3′ terminal of BcPV3.

Using RNA ligase-mediated RACE and classic RACE techniques, the terminal sequences of all RNA viruses were cloned, enabling the acquisition of their full-length viral genomes (Figure 4). Both BfPV1/KY-1 and BcPV3/KY-1V1 contained three dsRNA segments, encoding the RNA-dependent RNA polymerase, capsid protein, and a hypothetical protein, respectively. The terminal sequences of these two viruses exhibited conservation: BfPV1 featured a 5′-terminal conserved sequence “GCGCAAA” and a 3′-terminal sequence “AAUCC”, while BcPV3 had a 5′-terminal conserved sequence “GCGAAAUUU” and a 3′-terminal sequence “AUAG” (Figure 4A,B).

The nucleotide identities of dsRNA1 and dsRNA2 of BfPV1/KY-1 reached 99% and 96%, respectively, with the corresponding segments of Botryotinia fuckeliana partitivirus 1 (BfPV1) (accession numbers MN954881.1 and MN954882.1). However, the dsRNA3 of BfPV1/KY-1 differed from BfPV1 dsRNA3 and was not a defective fragment derived from dsRNA2 (Table 1). BLASTn analysis revealed that dsRNA1 of BcPV3/KY-1V1 shared 95–96% nucleotide identity (99% query coverage) with dsRNA1 of both BcPV3 and Sclerotinia sclerotiorum partitivirus 3 (MF444214.1, MN954884.1). Meanwhile, dsRNA2 of BcPV3/KY-1V1 showed 97% nucleotide identity (99% query coverage) with dsRNA2 of Sclerotinia sclerotiorum partitivirus 2 (MF444213.1), and 89% nucleotide identity (99% query coverage) with dsRNA2 of Botrytis cinerea partitivirus 3 (MN954883.1) (Table 1). Notably, dsRNA1 of Sclerotinia sclerotiorum partitivirus 3 and dsRNA2 of Sclerotinia sclerotiorum partitivirus 2 might originate from different genomic segments of the same virus.

### 3.3. Phylogenetic Position of Identified RNA Viruses

**Figure 5:**
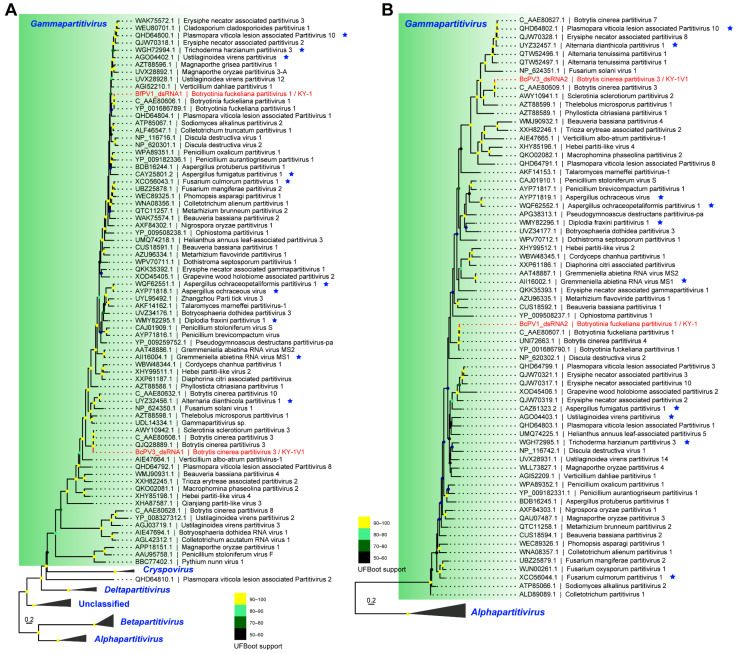
Phylogenetic analysis based on the RdRP and CP of the partitivirus in KY-1 and KY-1V1. (**A**) Phylogenetic tree of RdRP. (**B**) Phylogenetic tree of CP. The blue star in the figure indicates a partitivirus containing three genomic segments. The red font indicates the partitivirus discovered in this study. The best-fit substitution model (Q.pfam + F + R7 for RdRP, Q.pfam + F + I + R5 for CP) was selected based on the Bayesian information criterion (BIC) in ModelFinder. Ultrafast bootstrap support values from 10,000 replicates are shown near the nodes. The scale bar represents 0.2 substitutions per site.

Based on phylogenetic trees of RdRP and CP, BfPV1/KY-1 and BcPV3/KY-1V1 were in two distinct clades within the genus *Gammapartitivirus* (Figure 5). In the RdRP phylogenetic tree, BfPV1/KY-1 clustered with BfPV1, while BcPV3/KY-1V1 clustered with BcPV3 from *B. cinerea* and SsPV3 from *Sclerotinia sclerotiorum* (Figure 5A). In the CP phylogenetic tree, BfPV1/KY-1 clustered with BfPV1 and BcPV4, whereas BcPV3/KY-1V1 clustered with BcPV3 from *B. cinerea* and SsPV2 from *S. sclerotiorum* (Figure 5B).

**Figure 6:**
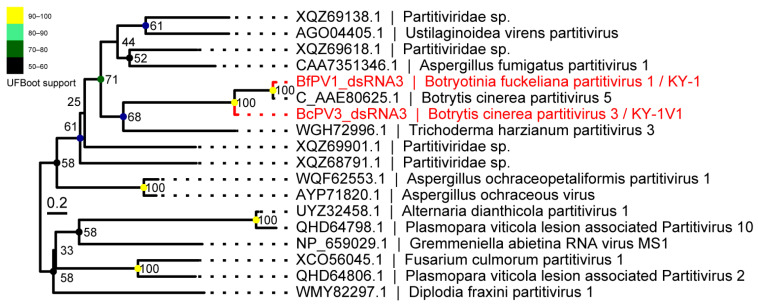
Phylogenetic analysis based on the HP (dsRNA3) of the partitivirus in KY-1 and KY-1V1. The best-fit substitution model (Q.yeast + R3) was selected based on the Bayesian information criterion (BIC) in ModelFinder. Ultrafast bootstrap support values from 10,000 replicates are shown near the nodes. The scale bar represents 0.2 substitutions per site.

The phylogenetic tree based on the hypothetical protein (HP) encoded by dsRNA3 showed that the HPs encoded by dsRNA3 of BfPV1/KY-1 and BcPV3/KY-1V1 clustered together with the hypothetical protein of BcPV5 (Figure 6).

The phylogenetic tree based on the RdRP of mitoviruses revealed that BcMV1, BcMV2, BcMV3, BcMV9, and SsMV3 clustered with their previously reported counterparts from *B. cinerea* or *S. sclerotiorum*. BcMV1, BcMV2/KY-1, and BcMV9/KY-1V1 clustered with their corresponding viruses BcMV1, BcMV2, and BcMV9 from *B. cinerea*. BcMV3/KY-1V1 clustered with the previously reported BcMV3 and SsMV35 from *S. sclerotiorum*. SsMV3/KY-1V1 clustered with the previously reported SsMV3 from *S. sclerotiorum*, MfMV3 from *Monilinia fructicola*, and BcMV7 from *B. cinerea* (Figure S1). Based on the phylogenetic tree of the RdRP domain of SsULV3/KY-1V1, it clustered with SsULV3 from *S. sclerotiorum* and BcULV4 from *B. cinerea*, belonging to the proposed virus family “Ambiguiviridae” (Figure S2). These results indicated that KY-1 and KY-1V1 harbor diverse RNA viruses, which show similarity to viruses previously reported in *B. cinerea* and *S. sclerotiorum*.

### 3.4. Discovery of Retroplasmids in B. cinerea

**Figure 7:**
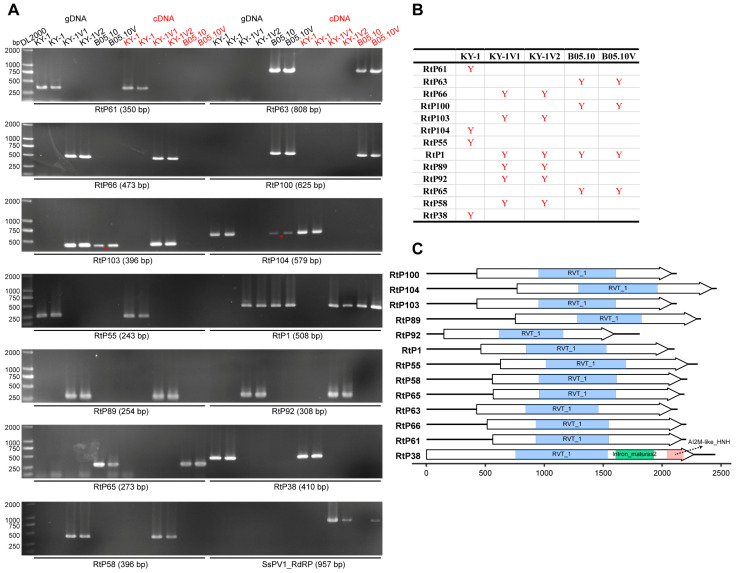
Detection of retroplasmids in *B. cinerea*. (**A**) PCR verification of retroplasmids in the KY-1 series strains and B05.10. The red asterisk indicates a weak band resulting from non-specific amplification. (**B**) Summary table of retroplasmid detection results in the KY-1 series strains and B05.10. The red “Y” in the figure indicates that the corresponding contig is detected in the strain. (**C**) The genome diagram of retroplasmids and group II intron. The reverse transcriptase domain (RVT_1) is shown in light blue. The type II intron maturase domain (Intron_maturas2) and AI2M-like HNH endonuclease domain (AI2M-like_HNH) within the group II intron are depicted in medium green and light pink, respectively.

Through virome analysis of the KY-1 serial strains and B05.10, in addition to the RNA viruses mentioned earlier, at least 13 contigs captured our attention because their BLAST results showed that the most similar sequences were a hypothetical protein of *Trichoderma harzianum* (GenBank accession AAF89327.2) or a reverse transcriptase of *Fusarium oxysporum* (GenBank accession AAD38504.1), rather than proteins from *B. cinerea* or its closely related species. Further analysis revealed that these contigs were retroplasmids encoding only reverse transcriptase. Unlike the reverse transcriptase-encoding transposons commonly found in fungal genomes, these retroplasmids only contained complete open reading frames (ORFs) longer than 100 aa when the mitochondrial genetic code was used for ORF prediction. This characteristic suggested that such retroplasmids shared similarities with mitoviruses that encode only RdRP, namely, they might parasitize in fungal mitochondria. PCR detection identified three retroplasmids in KY-1, six in KY-1V1 and KY-1V3, and four in the *B. cinerea* strain B05.10. These retroplasmids could be detected at both the RNA and DNA levels (Figure 7). KY-1V1 and KY-1V2 shared identical RNA virus and retroplasmid compositions (Figure 3 and Figure 7), suggesting they may share an identical genetic background. The contig RtP38, specifically detected in KY-1, also only revealed an ORF containing a reverse transcriptase domain when predicted using the mitochondrial genetic code. However, BLASTp analysis showed its highest similarity to a hypothetical protein of *Phyllosticta yuccae* (GenBank accession YP_010836049.1) with 99% coverage and 79% identity (Table S5). Domain-based analysis indicated that RtP38 was a group II intron probably integrated in the fungal mitochondrial genome (Figure 7C).

**Figure 8:**
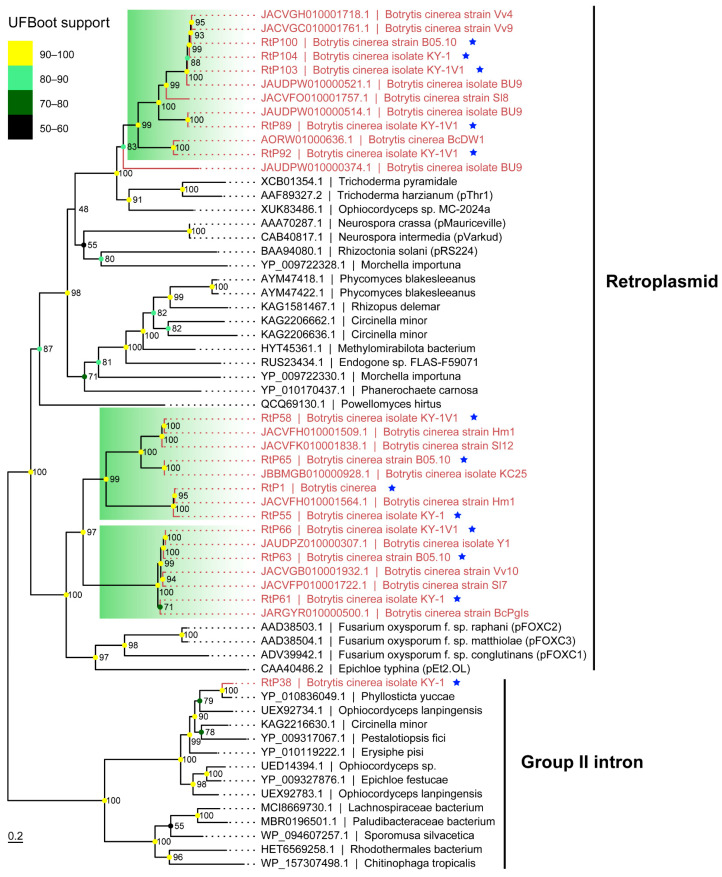
Phylogenetic analysis of retroplasmids in *B. cinerea*. The blue star in the figure indicates sequences identified in this study. The retroplasmids or group II intron of *B. cinerea* were highlighted in red font. The best-fit substitution model (VT + F + R5) was selected based on the Bayesian information criterion (BIC) in ModelFinder. Ultrafast bootstrap support values from 10,000 replicates are shown near the nodes.

Using the protein sequence of a retroplasmid (e.g., RtP55) as a query in a tBLASTn search against whole-genome shotgun contigs (limited to *Botrytis cinerea*; taxid: 40559), we obtained over 124 contigs (Table S6). These nucleotide sequences were translated into protein sequences using the fungal mitochondrial genetic code. After removing redundant sequences with thresholds of ≥95% coverage and ≥95% identity, 15 distinct retroplasmid sequences were retained. Phylogenetic analysis showed that retroplasmids from *B. cinerea* formed a large clade with those from other fungi. Within this clade, the *B. cinerea* retroplasmids formed three major subclades. Two were closely related to the pFOXC retroplasmids, while the third was related to the pThr1 retroplasmid from *T. harzianum* (Figure 8). This clustering pattern was further supported by both pairwise nucleotide and protein sequence identities (Figure S3). In contrast, RtP38 from KY-1 clustered within group II introns. Compared to KY-1, the retroplasmids in KY-1V1 showed closer phylogenetic relationships to those in B05.10. For example, RtP1 was detected in both KY-1V1 and B05.10 (Figure 7), and RtP63 and RtP58 in KY-1V1 were phylogenetically closer to RtP66 and RtP65 in B05.10, respectively (Figure 8).

## 4. Discussion

In this study, we identified three RNA viruses in KY-1 and seven in KY-1V1, and successfully cloned their full-length genomes. Among these, BfPV1 and BcPV3 described in this study contain three dsRNAs, with their dsRNA3 being a conserved RNA satellite among certain viruses in the genus *Gammapartitivirus*. Furthermore, we describe for the first time the discovery of 12 retroplasmids in *B. cinerea*, which reveals the tip of a hidden iceberg in the virosphere of *B. cinerea* and potentially other fungi.

While attempting to investigate the mechanism behind the previously reported phenotypic effects of SsPV1 on KY-1 [55], we unexpectedly discovered genetic background differences between the KY-1 and KY-1V1 strains, as well as variations in their RNA virus and retroplasmid compositions. Upon reviewing and reflecting on previous study [55], we consider the following speculation to be the most plausible explanation for our current results: the primary isolates obtained directly from a diseased blueberry fruit may have originally consisted of distinct *B. cinerea* strains. During transfection experiments with SsPV1, one of these strains was successfully transfected, yielding KY-1V1 and KY-1V2, while only the existing KY-1 strain was retained through subsequent subculturing of the primary isolate. Since the original primary isolates are no longer available, this hypothesis is difficult to verify. Nonetheless, our case serves as a reminder to mycovirologists of the importance of ensuring consistent and stable genetic backgrounds of fungal hosts during strain isolation and in studies of virus–host interactions. For most fungi, strains with consistent genetic background could be obtained by purification through single sexual or asexual spore. The reliability of this method has been recently supported by a study showing that although conidia of *B. cinerea* or ascospores of *S. sclerotiorum* are multinucleate, a full set of nuclear genetic material (i.e., chromosomes) is not in single nucleus than non-uniformly distributed among these nuclei [70]. However, single-spore isolation may lead to the loss of pre-existing viruses in the original fungal isolates, making this method unsuitable for viral diversity studies that require the examination of numerous fungal strains [20]. Although not the original intent of this study, our findings reveal a pitfall in mycovirology and remind researchers in the field to consider the importance of genetic background in fungus–virus systems.

The complete genomic sequences of BfPV1 and BcPV3, along with their RNA satellite (dsRNA3) are determined. Specifically, the dsRNA1 and dsRNA2 of BfPV1 are highly similar to the corresponding segments of BfPV1, which was named based on the teleomorph of *B. cinerea* (*Botryotinia fuckeliana*). Interestingly, the BfPV1 dsRNA3 submitted to GenBank by De Guido et al., which probably was isolated from Italy, is a defective fragment of dsRNA2. BfPV1 isolated from Italy or Spain by Ruiz-Padilla et al. was also lost or not obtained the dsRNA3 after re-analysis the viromic data [17]. However, Duan et al. misidentified the dsRNA3 of BfPV1 as a genomic segment of a novel partitivirus and classified it as Botrytis cinerea partitivirus 5, which was isolated from Israel [20]. BfPV1 identified in KY-1 was from United States. The dsRNA1 and dsRNA2 of BcPV3 reported in this study are consistent with the corresponding sequences of BcPV3 reported by Duan et al. and Ruiz-Padilla et al., but these two studies failed to identify the dsRNA3 of BcPV3, which is probably lost or not obtained in Israel and Spanish isolates [17,20]. The genomic characterization of BcPV3 in this study, combined with the findings of Duan et al. and Ruiz-Padilla et al., collectively confirmed that SsPV2 and SsPV3, reported by Mu et al. in *S. sclerotiorum* isolated from Australia, are probably two different segments of the same partitivirus [17,20,71]. Furthermore, the dsRNA3 was also not found in SsPV2 (or SsPV3) after a BLASTp search against all assembled contigs. By fully presenting the genomes of BfPV1 and BcPV3, the RNA satellites (dsRNA3) were found to be lost or not obtained in some isolates from different geographic regions. Additionally, the RdRP and CP phylogenetic tree put BfPV1 and BcPV3 in different clades, and dsRNA3 of BfPV1 and BcPV3 located in same clades in phylogenetic tree, implying the horizontal gene transfer event of the RNA satellite.

BcPV3, SsMV3, and SsULV3 carried by KY-1 and KY-1V1 share the high similarity with corresponding viruses from *S. sclerotiorum*, suggesting these viruses might have the potential for cross-species transmission. However, in confrontation assays between KY-1V1 and *S. sclerotiorum* strains 1980 and Ep-1PNA367, no successful viral transmission to *S. sclerotiorum* was observed. Nevertheless, studies by Deng et al. and Xiao et al. demonstrated that fungal viruses can indeed undergo horizontal transmission across species boundaries [55,72]. We speculated that transmission conditions or strain backgrounds in this experiment may have prevented successful transmission.

Researchers have made significant efforts to describe the diversity of RNA viruses in the virosphere [73,74]. For example, in fungi, an increasing number of fungal species have been found to harbor a rich variety of RNA viruses [62,75]. In addition to these RNA viruses, some viroid-like RNA elements have recently been discovered in diverse organisms (including fungi [76,77,78,79]) and environments (such as the human gut [80]). These viroids and viroid-like RNA elements sensu stricto differ from canonical viruses and are suggested to be classified into the perivirosphere [35]. We identified retroplasmids in strains KY-1, KY-1V1, and B05.10, representing the first report of such mitochondrial virus-like molecular parasites in *B. cinerea*. Retroplasmids are unique mitochondrial MGEs that are different from the other two class of fungal mitochondrial plasmids which are circular double-stranded DNA plasmids encoding DNA polymerase or linear double-stranded DNA plasmids encoding both DNA and RNA polymerases [81,82]. Retroplasmids rely on their encoded reverse transcriptase for replication via reverse transcription. Among these, the circular Mauriceville plasmid in *Neurospora crassa* and the linear pFOXC plasmids in *F. oxysporum* are well-studied representatives [36]. In this study, attempts to amplify retroplasmids in *B. cinerea* using reverse primers yielded no amplification products, suggesting that the retroplasmids in *B. cinerea* may have a linear structure similar to the pFOXC plasmids in *F. oxysporum* [38]. Currently, the full-length sequences of retroplasmids in *B. cinerea* remain to be cloned.

Since retroplasmids are localized in mitochondria, it is difficult to eliminate them through conventional methods (e.g., protoplast or conidial progeny) to study their effects on the host. Referring to studies in *Podospora* sp. where sexual reproduction generated progeny lacking mitochondrial plasmid [83], *B. cinerea* is a heterothallic ascomycete, and obtaining retroplasmid-free progeny would require crossing with a compatible *B. cinerea* strain of opposite mating type that lacks retroplasmids. Previous studies have shown that in *Neurospora* spp., retroplasmids may be associated with senescence and mitochondrial dysfunction [84,85,86]. This study also detected retroplasmids in the commonly used *B. cinerea* strain B05.10 and *B. cinerea* isolates in whole-genome shotgun database. Their potential impact on host biological characteristics warrants further investigation.

In addition to retroplasmids, we identified a group II intron in the KY-1 strain, designated as RtP38, in this study. The group II intron is absent in both KY-1V1 and B05.10 strains, and its distribution in *B. cinerea* appears to be less widespread than that of retroplasmids. For instance, in a tBLASTn search using the RtP38 protein sequence against the whole-genome shotgun (WGS) data of *B. cinerea* (taxid: 40559), only one contig (GenBank: JACVFJ010001546.1) from *B. cinerea* strain Rf1 R11546 exhibited high homology with RtP38 (100% coverage, 98% identity). Through re-sequencing of the KY-1 genome, we confirmed that RtP38 is integrated into the mitochondrial genome, located downstream of a NADH:ubiquinone oxidoreductase subunit 2 (chain N) gene, whereas retroplasmids were not integrated into the mitogenome. Correct splicing of fungal mitochondrial introns or proteins encoded by these introns (i.e., intron-encoded proteins) may influence mitochondrial gene expression or host phenotypes [87]. For example, group I-D introns might implicate in fungicide resistance to quinone outside inhibitors [88], and small molecule-mediated inhibition of group II intron splicing in *Candida parapsilosis* has been shown to suppress fungal growth [89,90]. Additionally, the hypovirulence of *Cryphonectria parasitica* strain KFC9 has been linked to the insertion of an ORF-less type A1 group II intron into the mitochondrial small-subunit ribosomal RNA (*rns*) gene [91]. Further investigation is warranted to elucidate the splicing mechanism of RtP38 in the KY-1 strain, its potential impact on the expression of adjacent genes, and whether its encoded protein influences the phenotype of *B. cinerea*. Such studies would contribute to a deeper understanding of how group II introns in fungal mitochondria affect phenotypic traits and ecological adaptation.

## 5. Conclusions

This study revealed that KY-1 and KY-1V1 are distinct strains of *B. cinerea* and harbor different RNA viruses. KY-1 contains the mitoviruses BcMV1 and BcMV2, along with the partitivirus BfPV1. KY-1V1 carries four mitoviruses (BcMV1, BcMV3, BcMV9, and SsMV3), one umbra-like virus (SsULV3), and two partitiviruses (BcPV3 and SsPV1). By cloning the complete genomes of the partitiviruses BfPV1 and BcPV3, this study identified a dsRNA3 segment as RNA satellites of these two viruses. Additionally, the study identified three retroplasmids in KY-1, six in KY-1V1, and four in B05.10, representing the first report of retroplasmids in *B. cinerea*.

## Figures and Tables

**Table 1:** Comparative analysis of RNA viruses identified in this study against NT database by BLASTn with megablast algorithm. The “n.a.” in the table indicates no significant hit in the BLASTn search.

Family	Query		Length (bp)	Hit Length	Hit Accession	Hit Title	Coverage (%)	Identity (%)
*Mitoviridae*	BcMV1		2808	2788	MT119677	Botrytis cinerea mitovirus 1 isolate BCS1_DN4879	99	98
	BcMV2/KY-1		2506	2486	PV443060	Botrytis cinerea mitovirus 2 isolate IBc-374	99	96
	BcMV3/KY-1V1		2895	2870	ON738336	Botrytis cinerea mitovirus 3 isolate VPS3	98	93
	BcMV9/KY-1V1		2737	2720	MT089704	Botrytis cinerea mitovirus 9 isolate BCS1_DN2958	99	96
	SsMV3/KY-1V1		2591	2588	NC_076556	Sclerotinia sclerotiorum mitovirus 3	100	93
“Ambiguiviridae”	SsULV3/KY-1V1		4003	3981	MT230952	Sclerotinia sclerotiorum umbra-like virus 3 isolate BCS17_DN25	92	97
*Partitiviridae*	BcPV1/ KY-1	dsRNA1	1795	1780	MN954881	Botryotinia fuckeliana partitivirus 1 isolate BCS3_DN4616 segment RNA1	99	99
				1793	AM491609	Botryotinia fuckeliana partitivirus 1, complete segment 1	100	98
		dsRNA2	1594	1597	MN954882	Botryotinia fuckeliana partitivirus 1 isolate BCI12_DN10399 segment RNA2	99	96
				1566	AM491610	Botryotinia fuckeliana partitivirus 1, complete segment 2	98	91
		dsRNA3	1298	n.a.	n.a.	No significant hit.	n.a.	n.a.
	BcPV3/ KY-1V1	dsRNA1	1784	1769	MF444214	Sclerotinia sclerotiorum partitivirus 3 isolate SsPV3 RdRP gene	99	95
				1762	MN954884	Botrytis cinerea partitivirus 3 isolate BCS4_DN10017 segment RNA1	99	96
		dsRNA2	1555	1547	MF444213	Sclerotinia sclerotiorum partitivirus 2 isolate SsPV2 coat protein gene	98	97
				1537	MN954883	Botrytis cinerea partitivirus 3 isolate BCS4_DN5031 segment RNA2	99	89
		dsRNA3	1260	n.a.	n.a.	No significant hit.	n.a.	n.a.

## Data Availability

The RNA-seq and DNA-seq data are available under BioProject accession PRJNA1348606. The sequences of all RNA viruses and retroplasmids have been deposited in GenBank under accessions PX521013–PX521037, and are also provided in Supplementary Materials.

## Supplementary Materials

The following supporting information can be downloaded at: https://www.mdpi.com/article/10.3390/v17121527/s1; Figure S1: Phylogenetic analysis of mitoviruses in *B. cinerea*; Figure S2: Phylogenetic analysis of SsULV3; Figure S3: Analysis of pairwise nucleotide and protein sequence identities identified in *B. cinerea*; Table S1: Primers for the amplification of multiple genes and microsatellite regions in *B. cinerea* strains; Table S2: Primers used for viral detection in strains such as KY-1; Table S3: Primers for the detection of retroplasmids in *B. cinerea* strains; Table S4: The primers used for cloning the termini of the virus in strains such as KY-1; Table S5: BLASTp analysis of retroplasmids identified in this study against NR database; Table S6: tBLASTn analysis of protein sequence of RtP55 against whole-genome shotgun contigs (wgs) database limited by *Botrytis cinerea* (taxid:40559); Supplementary text: Nucleotide sequences of RNA viruses, retroplasmids, and group II intron identified in this study.

## Abbreviations

The following abbreviations are used in this manuscript:

HP	Hypothetical protein
MGE	Mobile genetic element
ORF	Open reading frame
RACE	Rapid amplification of cDNA ends
RdRP	RNA-dependent RNA polymerase
RtP	Retroplasmid
