# Supplementary material for: The Viruses of Botrytis cinerea and Beyond: Molecular Characterization of RNA Viruses and Retroplasmids
Source: Viruses. 2025 Nov 21;17(12):1527. doi: 10.3390/v17121527 (PMC12737674; doi:10.3390/v17121527)
Supplement: Supplementary file 1 [file viruses-17-01527-s001.zip › viruses-3983138-supplementary/Supplementary figures S1-S3.pdf]

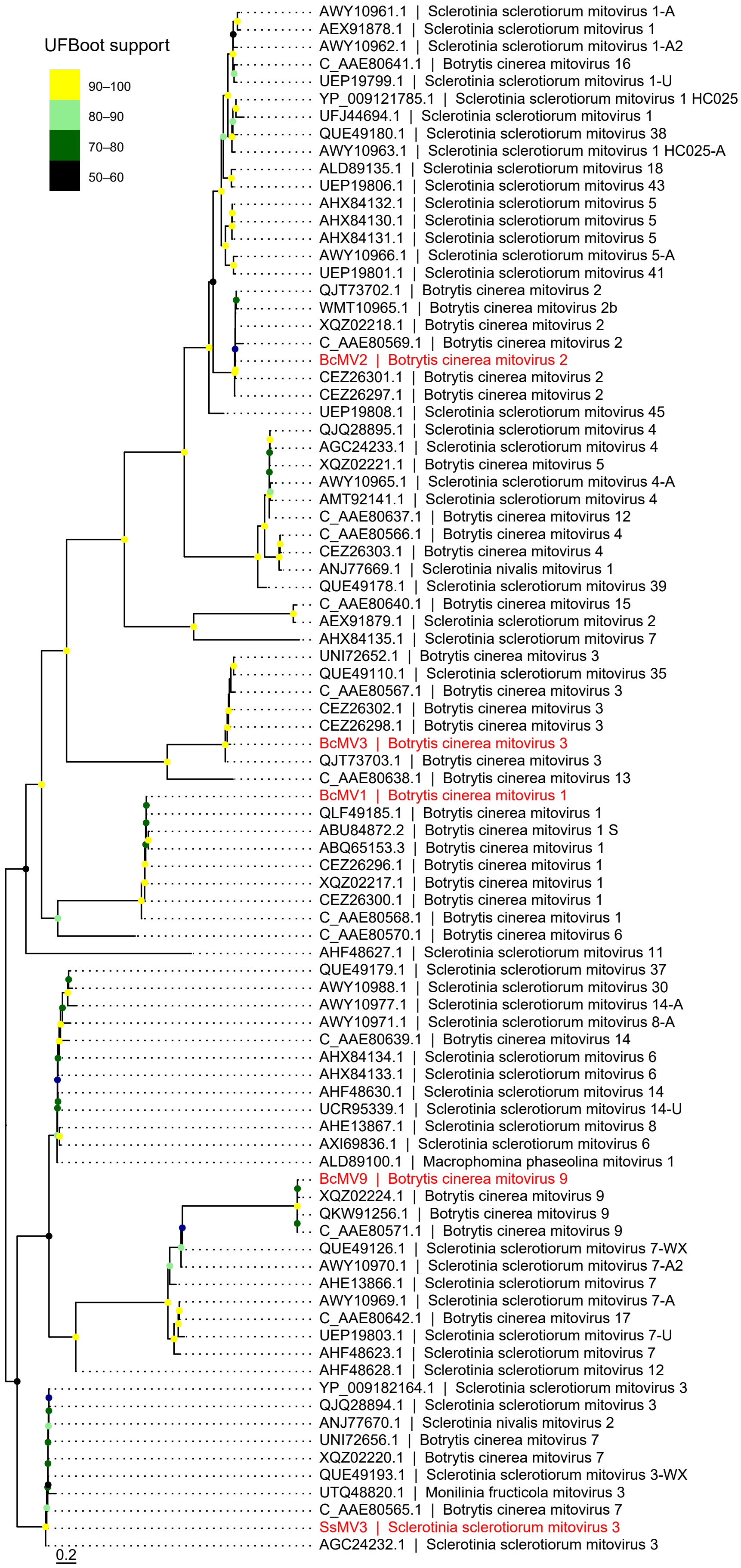


Figure S1. Phylogenetic analysis of mitoviruses in *B. cinerea*. The viruses cloned in this study were highlight in red. The best-fit substitution model (WAG+F+R5) was selected based on the Bayesian information criterion (BIC) in ModelFinder. Ultrafast bootstrap support values from 10,000 replicates are shown near the nodes.

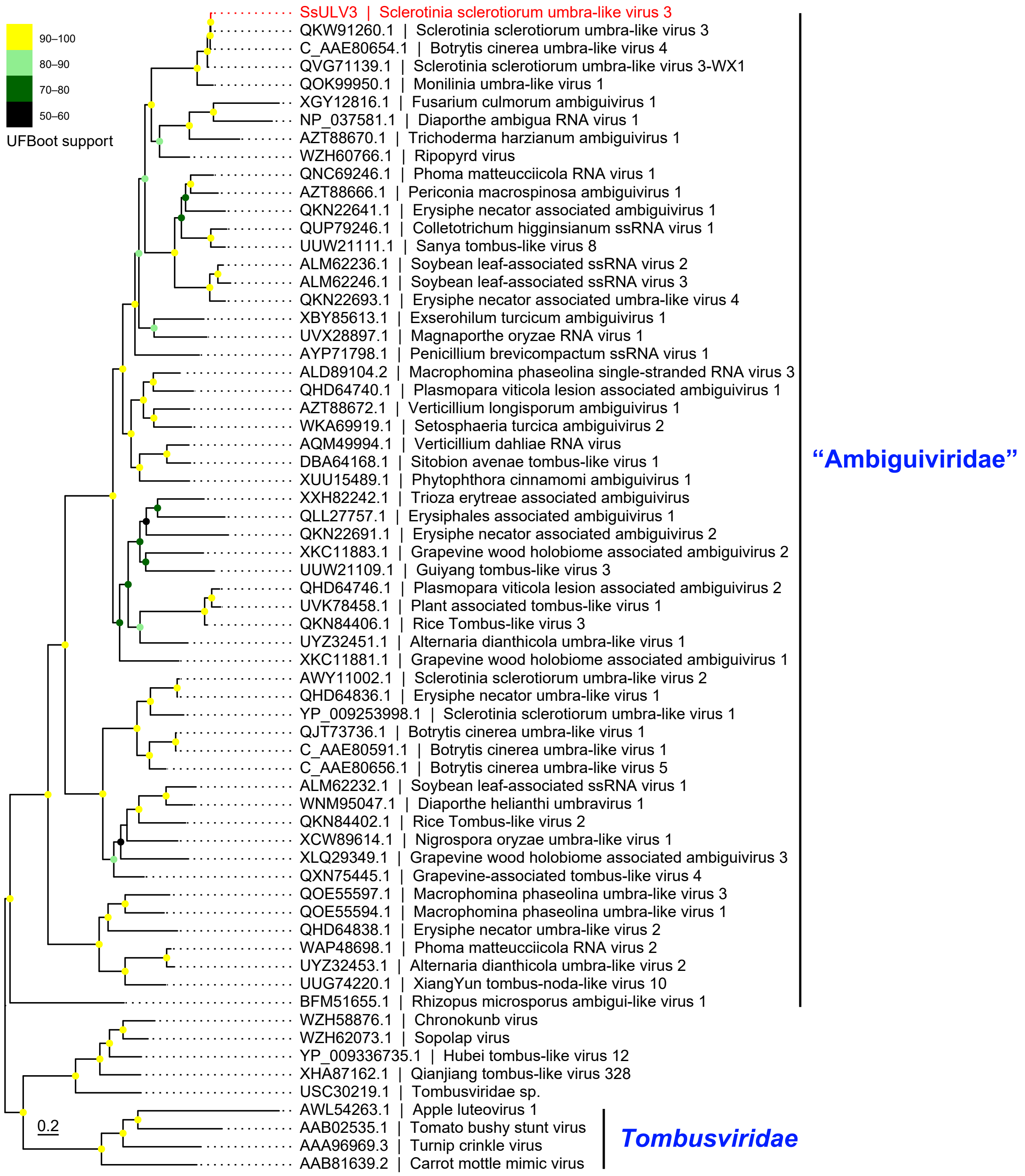


Figure S2. Phylogenetic analysis of SsULV3. The virus cloned in this study was highlight in red. The best-fit substitution model (WAG+F+R5) was selected based on the Bayesian information criterion (BIC) in ModelFinder. Ultrafast bootstrap support values from 10,000 replicates are shown near the nodes.

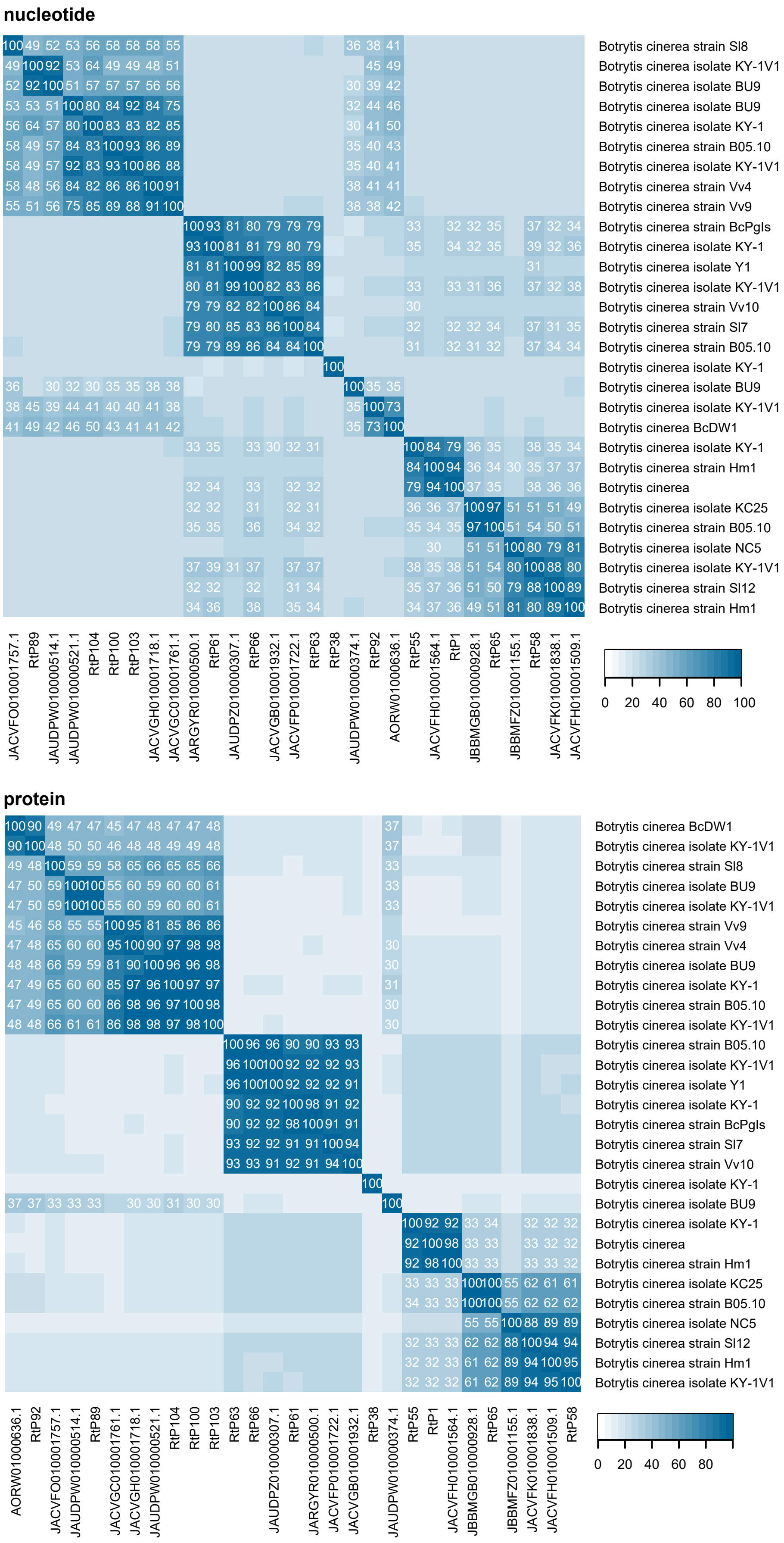


Figure S3. Analysis of pairwise nucleotide and protein sequence identities identified in *B. cinerea*. The analysis was performed using Clustal Omega (v1.2.4), and the results were visualized as a heatmap using the heatmap.2 function from the gplots package in R.
