# Supplementary material for: The Viruses of Botrytis cinerea and Beyond: Molecular Characterization of RNA Viruses and Retroplasmids
Source: Viruses. 2025 Nov 21;17(12):1527. doi: 10.3390/v17121527 (PMC12737674; doi:10.3390/v17121527)
Supplement: Supplementary file 1 [file viruses-17-01527-s001.zip › viruses-3983138-supplementary/Supplementary tables S1-S6.pdf]

Table S1. Primers for the amplification of multiple genes and microsatellite regions in *B. cinerea* strains.

Name	Sequence	Note/Product size
G3PDH-F	ATTGACATCGTCGCTGTCAACGA	973 bp
G3PDH-R	ACCCCACTCGTTGTCGTACCA	973 bp
HSP60-F	CAACAATTGAGATTTGCCCACAAG	1096 bp
HSP60-R	GATGGATCCAGTGGTACCGAGCAT	1096 bp
RPB2-F	GATGATCGTGATCATTTCGG	1220 bp
RPB2-R	CCCATAGCTTGCTTACCCAT	1220 bp
ITS1	TCCGTAGGTGAACCTGCGG	520 bp
ITS4	TCCTCCGCTTATTGATATGC	520 bp
Bc1F	AGGGAGGGTATGAGTGTGTA	247 bp
Bc1R	TTGAGGAGGTGGAAGTTGTA	247 bp
Bc2F	CATACACGTATTTCTTCCAA	165 bp
Bc2R	TTTACGAGTGTTTTTGTTAG	165 bp
Bc3F	GGATGAATCAGTTGTTTGTG	225 bp
Bc3R	CACCTAGGTATTTCCTGGTA	225 bp
Bc4F	CATCTTCTGGGAACGCACAT	125 bp
Bc4R	ATCCACCCCCAAACGATTGT	125 bp
Bc5F	CGTTTTCCAGCATTTCAAGT	159 bp
Bc5R	CATCTCATATTCGTTCCTCA	159 bp
Bc6F	ACTAGATTCGAGATTCAGTT	122 bp
Bc6R	AAGGTGGTATGAGCGGTTTA	122 bp
Bc7F	CCAGTTTCGAGGAGGTCCAC	121 bp
Bc7R	GCCTTAGCGGATGTGAGGTA	121 bp
Bc9F	CTCGTCATAACCACGCAGAT	160 bp
Bc9R	GCAAGGTCTCGATGTCGATC	160 bp
Bc10F	TCCTCTTCCCTCCCATCAAC	181 bp
Bc10R	GGATCTGCGTGGTTATGACG	181 bp

Table S2. Primers used for viral detection in strains such as KY-1.

Name	Sequence	Note/Product size
BcMV1-F	GGAGAAGATACCTAAGCAGAT	BcMV1 detection, 487 bp
BcMV1-R	GGCGTAGCAGACATTACA	BcMV1 detection, 487 bp
BcMV2-F	CAAGATACATAAGTGGACAACC	BcMV2 detection, 597 bp
BcMV2-R	AGTAGTCTACCATAGCAATAGC	BcMV2 detection, 597 bp
BcMV3-F	ACCAATATGTGACCGTGAT	BcMV3 detection
BcMV3-R	CAGCAGCGACAGATAGAT	BcMV3 detection
BcMV-F	ACCAGCCATCTTCATCAAT	BcMV9 detection, 842 bp
BcMV-R	TCGCCATTACTCATCATACT	BcMV9 detection, 842 bp
SsMV3-F	TTGTCCTTAACCACCTTGT	SsMV3 detection, 754 bp
SsMV3-R	CCACGGATAGACTACCAATA	SsMV3 detection, 754 bp
MoRV-F	TATGTCGTGGTGGAGTCT	SsULV-3 detection
MoRV-R	TGTGGAGGTAGTGTGGAA	SsULV-3 detection
BfPV-RP1	ACGCTTGTACACCGAATGGT	869 bp, BfPV1 dsRNA1, RdRP
BfPV-RP2	ACTCCTACGCTAGGTGTCGT	869 bp, BfPV1 dsRNA1, RdRP
BfPV-CP1	CCAGAAAACACAGCCGTTGG	688 bp, BfPV1 dsRNA2, CP
BfPV-CP2	TTCCGGCTCTCGGAACTAGA	688 bp, BfPV1 dsRNA2, CP
UvPVS4-1	TTCGCTCCCTTCCAATCCAC	511 bp, BfPV1 dsRNA3
UvPVS4-2	AGTGGTTCTATTGTCGGGCG	511 bp, BfPV1 dsRNA3
BcPV2_RP_F	CCGTTTCTCGGTGATAACC	405 bp, SsPV2, BcPV3, dsRNA1
BcPV2_RP_R	AACCTCTTCCCACTCAAGC	405 bp, SsPV2, BcPV3, dsRNA1
PdCP1	TGCAGAGACGGTGAAAGCAT	655 bp, SsPV2, BcPV3, dsRNA2
PdCP2	GAAGACACCAGAAAGCGGGA	655 bp, SsPV2, BcPV3, dsRNA2
UvPV-F	TGTTGTGGAGTAGTAGGAAG	524 bp, SsPV2, BcPV3, dsRNA3
UvPV-R	AAGAAGTCGGCTAGTGTTAA	524 bp, SsPV2, BcPV3, dsRNA3

Table S3. Primers for the detection of retroplasmids in *B. cinerea* strains.

Name	Sequence	Note/Product size
RtP55-F	GGAAGTGAGAGGTGTCAGAGT	243 bp
RtP55-R	ACAGGTCATTACAGGCATAGGA	243 bp
contig1-F	AATTGTGAACACACTAATGAC	508 bp
contig1-R	TCATGCCGTTTGCGTTCCTA	508 bp
RtP58-F	GCCATTCAGCAAGCAATTAGAT	396 bp
RtP58-R	CTAGCCTCAGATCAAGCATCAT	396 bp
RtP61-F	CGGAGGCGGAATATACCATC	350 bp
RtP61-R	GGTGTCTTGACTTGTTGAATCC	350 bp
RtP63-F	GAATAACATGCGTCGGCCAACG	808 bp
RtP63-R	GATTTCCACATAAGCTGCTAAC	808 bp
RtP66-F	GAGAACATTCTTGCTACCTGAG	473 bp
RtP66-R	CCAGTACGTCCAAATGTCTTAG	473 bp
RtP65-F	GCAAGGCGAAGGAAGATAG	273 bp
RtP65-R	ATCAAGGTAGACAGGATTACTC	273 bp
RtP89-F	CTCTCCAAGTTGGCGTATAGC	254 bp
RtP89-R	TTCGGAAGTTGTCTGGCATTC	254 bp
RtP92-F	TCAAGGAGCACCAACATCT	308 bp
RtP92-R	CCATTAAGTCATCACCAAGTCT	308 bp
RtP100-F	ACAGACCAGGACGAGGTACA	625 bp
RtP100-R	TCGCATCAAACTCCTGCGTA	625 bp
RtP103-F	GATACCGACCAGGACGAGGAA	396 bp
RtP103-R	CTGTTGCGAAGTGTGGAGTCA	396 bp
RtP104-F	TCACCCGCATGACGTGTATC	579 bp
RtP104-R	TTCGGCTTGGCTCAGATAGC	579 bp
RtP38-F	CGTGAAGAGTGAACAACCTAAG	410 bp
RtP38-R	CCGAACTTATACCGTCCAGAG	410 bp

Table S4. The primers used for cloning the termini of the virus in strains such as KY-1.

Name	Sequence	Note/Product size
PC2	CCGAATTCCCGGGATCC	hot-start and high Tm (57 °C)/Ta to avoid PC2 dimer
PC3-T7 loop	pGGATCCCGGGAATTCGGTAATACGACTCACTATATTTTTATAGTGAGTCGTATTA	5' terminal P modification
RT-adaptor +A tail	GAGGACTCGAGCTCAAGCATGCATTTTTTTTTTTTTT	RACE
RT-adaptor +C tail	GAGGACTCGAGCTCAAGCATGCATGGGGGGGGGGGGGG	RACE
PCR primer 1 － tail adaptor	GAGGACTCGAGCTCAAGC	RACE
BcMV1_5R1	TATTGTTTGGTCTTCGTGACCG	407 bp
BcMV1_5R2	TCTTCGTGACCGACAGTCT	397 bp
BcMV1_3F1	TTGCGAAATGCTTACTACCC	683 bp
BcMV1_3F2	GAATTGGTTGAATAATAGGGACA	409 bp
BcMV2_5R1	TCGTGAGGCAGGGGAATAGA	311 bp
BcMV2_5R2	CCACGCGTACCAAACATTCC	274 bp
BcMV2_3F1	AAGGTGGCTGAGAAATCAGG	540 bp
BcMV2_3F2	AGATGTTAGTACGCCATCCCA	455 bp
BcMV3_5R1	GCCCTATTAGCAAGACCCCG	598 bp
BcMV3_5R2	TGTATGGGTTCTCCTTCGTGG	302 bp
BcMV3_3F1	AAGTGCTGAACGGTGAGTCT	649 bp
BcMV3_3F2	AGAAGCAAACATGATGGACT	513 bp
BcMV9_5R1	GACATTGGTATGCCAGTTCCT	327 bp
BcMV9_5R2	CAACCATAGTCTCCACTTCTAC	265 bp
BcMV9_3F1	GGTGGATCTAGTAGTCCTTC	293 bp
BcMV9_3F2	TGAGTAAAACCTCTGTGGAGC	196 bp
BfPV1_S1_5R1	GCCTTGTACATGCCAGGAGT	393 bp
BfPV1_S1_5R2	AAGTTGGGCGACCTGTAGTC	234 bp
BfPV1_S1_3F1	TATCCGTGTCCTGAGGAGGG	271 bp
BfPV1_S1_3F2	GGAGGGGTGGTTCTCCAAAG	257 bp
BfPV1_S2_5R1	CTCCGCATGTACCCGAAACT	492 bp

Name	Sequence	Note/Product size
BfPV1_S2_5R2	ACACCAGTTGCGAAGGTCAT	314 bp
BfPV1_S2_3F1	CCAACGGCTGTGTTTTCTGG	327 bp
BfPV1_S2_3F2	GACACAGAGAGCCACCGAAT	265 bp
BfPV1_S3_5R1	TGCGCTAAGATGGGTGAGTT	340 bp
BfPV1_S3_5R2	CGTCAACAGGGTAACAGCCT	317 bp
BfPV1_S3_3F1	ATCCCTTGTCCATCCAAGGC	337 bp
BfPV1_S3_3F2	AGACCAAATCGCCCGACAAT	277 bp
BcPV3_S1_5R1	AACCTCTTCCCACTCAAGCG	530 bp
BcPV3_S1_5R2	CGGGTGAAACCGTAAATGCG	355 bp
BcPV3_S1_3F1	CTTATCGCAATCAACGCGCA	474 bp
BcPV3_S1_3F2	TCCAACGGATGGCACTTTCA	290 bp
BcPV3_S2_5R1	ACTTACCGGGTTGAGGGGTA	316 bp
BcPV3_S2_5R2	AAGCCTTTGGCCTGTTGTCT	215 bp
BcPV3_S2_3F1	CGAAAACGAGCAACCTACGC	481 bp
BcPV3_S2_3F2	GTGTTGCCAATCGCTCACAA	438 bp
BcPV3_S3_5R1	GCGCGGGTAAATCGATCCTT	429 bp
BcPV3_S3_5R2	TCTTGTCATGCAAGTCGGGG	323 bp
BcPV3_S3_3F1	GTTCACTCCCCGGTAATGTCA	548 bp
BcPV3_S3_3F2	TCAACCGACAACCCTTCGAT	407 bp
SsMV3_5R1	CTAAGAGCCGCAAATGCACC	421 bp
SsMV3_5R2	CATTGCTAAAACGGTCCGCTTG	402 bp
SsMV3_3F1	CAGCAGAAGGACTAGCATCG	663 bp
SsMV3_3F2	CGGGATTTAGGAGGACAACG	406 bp
SsULV3_5R1	CACGCCGTGGTTGTGAAG	614 bp
SsULV3_5R2	ACGTCCCCCAGTGATTTAAC	329 bp
SsULV3_3F1	AAATCTCGCGCCGATAGTGA	472 bp
SsULV3_3F2	CCTCCGCCAACTAGGATTCG	330 bp

Table S5. BLASTp analysis of retroplasmids identified in this study against NR database.

Query	Accession	Query length (aa)	Hit length (aa)	Hit accession	Hit title	Query coverage (%)	Identity (%)	E-value
RtP1	PX521025	530	527	AAD38503	reverse transcriptase [Fusarium oxysporum f. sp. raphani]	81	31	3.88E-46
RtP55	PX521026	530	527	AAD38504	reverse transcriptase [Fusarium oxysporum f. sp. matthiolae]	82	30	5.84E-44
RtP89	PX521027	513	569	XUK83486	hypothetical protein [Ophiocordyceps sp. MC-2024a]	94	35	4.28E-99
RtP92	PX521028	481	569	XUK83486	hypothetical protein [Ophiocordyceps sp. MC-2024a]	60	36	2.39E-57
RtP61	PX521029	534	527	AAD38504	reverse transcriptase [Fusarium oxysporum f. sp. matthiolae]	58	32	1.07E-38
RtP63	PX521030	551	527	AAD38504	reverse transcriptase [Fusarium oxysporum f. sp. matthiolae]	70	29	5.59E-38
RtP66	PX521031	550	527	AAD38504	reverse transcriptase [Fusarium oxysporum f. sp. matthiolae]	65	30	3.46E-38
RtP100	PX521032	551	569	XUK83486	hypothetical protein [Ophiocordyceps sp. MC-2024a]	85	36	2.05E-97
RtP103	PX521033	551	569	XUK83486	hypothetical protein [Ophiocordyceps sp. MC-2024a]	85	36	5.95E-97
RtP104	PX521034	551	569	XUK83486	hypothetical protein [Ophiocordyceps sp. MC-2024a]	85	36	8.80E-98
RtP65	PX521035	528	527	AAD38503	reverse transcriptase [Fusarium oxysporum f. sp. raphani]	87	31	1.21E-57
RtP58	PX521036	534	527	AAD38503	reverse transcriptase [Fusarium oxysporum f. sp. raphani]	85	30	6.80E-47
RtP38	PX521037	737	753	YP_010836049	hypothetical protein QLP54_mgp17 [Phyllosticta yuccae]	99	79	0

Table S6. tBLASTn analysis of protein sequence of RtP55 against whole-genome shotgun contigs (wgs) database limited by *Botrytis cinerea* (taxid:40559).

Description	Max Score	Total Score	Query Cover	E value	Per. ident	Acc. Len	Accession
Botrytis cinerea isolate NC5 NODE_936_length_2122_cov_120.112240	961	961	1	0	90.21	2122	JBBMFZ010000935.1
Botrytis cinerea strain Vv14 G142004	961	961	1	0	90.21	1921	JACVFX010002004.1
Botrytis cinerea strain Vv3 G31186	958	958	1	0	89.83	2157	JACVGL010001186.1
Botrytis cinerea strain Sl7 T71790	958	958	1	0	89.83	1834	JACVFP010001790.1
Botrytis cinerea strain Hm1 H11564	958	958	1	0	89.83	1750	JACVFH010001564.1
Botrytis cinerea strain Vv12 G121850	957	957	1	0	89.45	1914	JACVFZ010001850.1
Botrytis cinerea strain Sl2 T21477	956	956	1	0	89.45	2199	JACVFT010001477.1
Botrytis cinerea strain Sl5 T51758	956	956	1	0	89.45	2064	JACVFR010001758.1
Botrytis cinerea strain Sl4 T41720	956	956	1	0	89.45	1948	JACVFS010001720.1
Botrytis cinerea strain Sl9 T9521	956	956	1	0	89.45	2184	JACVFN010000521.1
Botrytis cinerea strain Sl11 T111750	956	956	1	0	89.27	1946	JACVFL010001750.1
Botrytis cinerea strain Sl8 T81755	955	955	1	0	89.45	1915	JACVFO010001755.1
Botrytis cinerea isolate NC7 NODE_946_length_2175_cov_168.763208	954	954	1	0	89.27	2175	JBBMGA010000945.1
Botrytis cinerea isolate Y1 Y1_SCAF__301	954	954	1	0	89.27	2135	JAUDPZ010000301.1
Botrytis cinerea isolate R23 R23_SCAF__414	954	954	1	0	89.27	2119	JAUDPY010000414.1
Botrytis cinerea isolate I9 I9_SCAF__322	954	954	1	0	89.27	2073	JAUDPX010000322.1
Botrytis cinerea isolate BU9 BU9_SCAF__492	954	954	1	0	89.27	2134	JAUDPW010000492.1
Botrytis cinerea strain Vv6 G61764	954	954	1	0	89.27	2127	JACVGF010001764.1
Botrytis cinerea strain Sl12 T121885	953	953	1	0	89.43	1648	JACVFK010001885.1
Botrytis cinerea isolate B5 B5_SCAF__373	950	950	1	0	89.08	2168	JAUDPV010000373.1

Description	Max Score	Total Score	Query Cover	E value	Per. ident	Acc. Len	Accession
Botrytis cinerea strain Vv7 G71939	949	949	1	0	89.27	1836	JACVGE010001939.1
Botrytis cinerea strain Rf2 R206827	883	883	0.92	0	89.98	1844	JACVFI010006827.1
Botrytis cinerea isolate KC25 NODE_1018_length_1432_cov_63.783588	833	833	0.87	0	89.78	1432	JBBMGB010001017.1
Botrytis cinerea strain Vv10 G102123	724	724	0.75	0	91.18	1253	JACVGB010002123.1
Botrytis cinerea strain Rf1 R11776	524	524	0.53	0	91.76	837	JACVFJ010001775.1
Botrytis cinerea isolate KC25 NODE_929_length_2057_cov_23.136364	271	271	0.9	2E-81	39.1	2057	JBBMGB010000928.1
Botrytis cinerea strain Vv12 G121874	259	259	0.73	3E-77	42.21	1820	JACVFZ010001874.1
Botrytis cinerea strain Vv4 G41714	258	258	0.73	4E-76	41.96	2070	JACVGH010001714.1
Botrytis cinerea strain Sl7 T71721	258	258	0.72	6E-76	42.16	2200	JACVFP010001721.1
Botrytis cinerea strain Hm1 H11509	254	254	0.72	9E-75	42.16	2138	JACVFH010001509.1
Botrytis cinerea strain Vv6 G61850	249	249	0.72	3E-74	41.9	1696	JACVGF010001850.1
Botrytis cinerea isolate R23 R23_SCAF__417	251	251	0.72	6E-74	41.65	1977	JAUDPY010000417.1
Botrytis cinerea isolate Y1 Y1_SCAF__304	251	251	0.72	8E-74	41.65	2009	JAUDPZ010000304.1
Botrytis cinerea strain Sl11 T111770	249	249	0.72	1E-73	41.65	1811	JACVFL010001770.1
Botrytis cinerea strain Sl8 T81790	248	248	0.72	1E-73	41.65	1780	JACVFO010001790.1
Botrytis cinerea strain Vv7 G71883	251	251	0.72	2E-73	41.65	2079	JACVGE010001883.1
Botrytis cinerea isolate W18 W18_SCAF__325	249	249	0.72	2E-73	41.65	1818	JATABW010000325.1
Botrytis cinerea strain Sl12 T121838	249	249	0.72	2E-73	41.65	1854	JACVFK010001838.1
Botrytis cinerea isolate NC7 NODE_940_length_2218_cov_77.103098	251	251	0.72	3E-73	41.65	2218	JBBMGA010000939.1
Botrytis cinerea strain Sl2 T21512	248	248	0.72	3E-73	41.39	1882	JACVFT010001512.1
Botrytis cinerea strain Sl4 T41718	249	249	0.72	4E-73	41.65	1958	JACVFS010001718.1

Description	Max Score	Total Score	Query Cover	E value	Per. ident	Acc. Len	Accession
Botrytis cinerea strain Vv3 G31195	249	249	0.72	5E-73	41.39	2075	JACVGL010001195.1
Botrytis cinerea isolate B5 B5_SCAF__374	248	248	0.72	2E-72	41.65	2137	JAUDPV010000374.1
Botrytis cinerea strain Sl5 T51750	248	248	0.72	2E-72	41.39	2077	JACVFR010001750.1
Botrytis cinerea strain Sl9 T9518	248	248	0.72	2E-72	41.65	2209	JACVFN010000518.1
Botrytis cinerea strain Vv14 G141931	248	248	0.72	3E-72	41.39	2197	JACVFX010001931.1
Botrytis cinerea strain Vv10 G102646	232	232	0.25	8E-72	87.02	658	JACVGB010002646.1
Botrytis cinerea strain Sl8 T81737	234	234	0.69	3E-67	39.47	2019	JACVFO010001737.1
Botrytis cinerea strain Sl9 T9520	234	234	0.63	4E-67	41.04	2189	JACVFN010000520.1
Botrytis cinerea strain Vv15 G151790	229	229	0.67	9E-67	39.19	1584	JACVFW010001790.1
Botrytis cinerea strain Sl7 T71722	233	233	0.69	2E-66	39.47	2190	JACVFP010001722.1
Botrytis cinerea strain Sl4 T41775	228	228	0.63	2E-66	40.75	1668	JACVFS010001775.1
Botrytis cinerea isolate KC25 NODE_932_length_2052_cov_61.038558	231	231	0.69	5E-66	39.58	2052	JBBMGB010000931.1
Botrytis cinerea strain Vv7 G71856	231	231	0.72	6E-66	38.36	2195	JACVGE010001856.1
Botrytis cinerea isolate NC5 NODE_984_length_1663_cov_109.163557	227	227	0.69	7E-66	39.47	1663	JBBMFZ010000983.1
Botrytis cinerea strain Sl11 T111771	228	228	0.63	7E-66	40.75	1798	JACVFL010001771.1
Botrytis cinerea strain Vv12 G121797	231	231	0.71	7E-66	39.33	2189	JACVFZ010001797.1
Botrytis cinerea strain Vv6 G61762	230	230	0.7	8E-66	39.37	2131	JACVGF010001762.1
Botrytis cinerea strain Rf2 R206630	228	228	0.67	1E-65	39.19	1906	JACVFI010006630.1
Botrytis cinerea strain Vv4 G41698	230	230	0.7	1E-65	39.37	2197	JACVGH010001698.1
Botrytis cinerea strain Vv10 G101932	227	227	0.69	1E-65	39.47	1768	JACVGB010001932.1
Botrytis cinerea strain Sl2 T21493	228	228	0.63	3E-65	40.75	2063	JACVFT010001493.1
Botrytis cinerea strain Sl5 T51757	228	228	0.63	3E-65	40.75	2067	JACVFR010001757.1

Description	Max Score	Total Score	Query Cover	E value	Per. ident	Acc. Len	Accession
Botrytis cinerea strain Sl12 T121776	228	228	0.63	3E-65	40.75	2090	JACVFK010001776.1
Botrytis cinerea strain Vv14 G141929	227	227	0.69	2E-64	39.47	2200	JACVFX010001929.1
Botrytis cinerea isolate Y1 Y1_SCAF__307	223	223	0.7	4E-64	39.11	1778	JAUDPZ010000307.1
Botrytis cinerea isolate R23 R23_SCAF__416	223	223	0.7	2E-63	39.11	2009	JAUDPY010000416.1
Botrytis cinerea isolate I9 I9_SCAF__319	223	223	0.7	3E-63	39.11	2132	JAUDPX010000319.1
Botrytis cinerea strain BcPgIs-3 scaffold_249	224	224	0.69	3E-63	39.41	2182	JBGCUU010000249.1
Botrytis cinerea isolate NC7 NODE_944_length_2176_cov_159.238095	223	223	0.7	4E-63	39.11	2176	JBBMGA010000943.1
Botrytis cinerea strain BcPgIs contig_500	224	448	0.69	2E-61	39.41	4313	JARGYR010000500.1
Botrytis cinerea isolate I9 I9_SCAF__327	217	217	0.57	4E-61	43.56	1962	JAUDPX010000327.1
Botrytis cinerea isolate NC5 NODE_1073_length_1227_cov_65.468430	174	174	0.41	2E-47	43.84	1227	JBBMFZ010001072.1
Botrytis cinerea strain Rf2 R214085	134	134	0.31	7E-35	44.51	634	JACVFI010014083.1
Botrytis cinerea isolate KC25 NODE_1452_length_476_cov_92.907363	124	124	0.13	8E-32	87.14	476	JBBMGB010001449.1
Botrytis cinerea isolate NC5 NODE_1156_length_963_cov_70.567181	82	82	0.3	2E-15	38.1	963	JBBMFZ010001155.1
Botrytis cinerea isolate NC5 NODE_1071_length_1236_cov_92.371719	79.7	79.7	0.53	3E-14	30.03	1236	JBBMFZ010001070.1
Botrytis cinerea isolate Y1 Y1_SCAF__309	79.7	79.7	0.53	5E-14	30.03	1580	JAUDPZ010000309.1
Botrytis cinerea isolate BU9 BU9_SCAF__567	79.7	79.7	0.53	5E-14	30.03	1581	JAUDPW010000567.1
Botrytis cinerea isolate I9 I9_SCAF__331	79.7	79.7	0.53	5E-14	30.03	1587	JAUDPX010000331.1
Botrytis cinerea strain Sl8 T81757	79.7	79.7	0.59	5E-14	28.53	1912	JACVFO010001757.1
Botrytis cinerea BcDW1 contig_636	79.7	79.7	0.53	6E-14	30.03	1915	AORW01000636.1

**Description**	**Max Score**	**Total Score**	**Query Cover**	**E value**	**Per. ident**	**Acc. Len**	**Accession**
Botrytis cinerea isolate NC7 NODE_206_length_67554_cov_35.095868	79.7	79.7	0.53	1E-13	30.03	67554	JBBMGA010000205.1
Botrytis cinerea isolate BU9 BU9_SCAF__374	77.8	155	0.44	3E-13	30.12	4912	JAUDPW010000374.1
Botrytis cinerea strain Vv6 G61891	76.3	76.3	0.59	5E-13	27.4	1499	JACVGF010001891.1
Botrytis cinerea isolate BU9 BU9_SCAF__630	74.3	74.3	0.52	2E-12	27.3	1343	JAUDPW010000630.1
Botrytis cinerea strain Vv9 G91761	74.7	74.7	0.62	2E-12	27.44	1740	JACVGC010001761.1
Botrytis cinerea strain Sl4 T41693	74.7	74.7	0.52	2E-12	27.3	2055	JACVFS010001693.1
Botrytis cinerea strain Sl7 T71737	74.7	74.7	0.52	2E-12	27.3	2097	JACVFP010001737.1
Botrytis cinerea isolate BU9 BU9_SCAF__521	74.3	74.3	0.52	3E-12	26.99	1825	JAUDPW010000521.1
Botrytis cinerea isolate NC7 NODE_983_length_1878_cov_90.104772	74.3	74.3	0.52	3E-12	26.99	1878	JBBMGA010000982.1
Botrytis cinerea isolate R23 R23_SCAF__419	74.3	74.3	0.52	3E-12	26.99	1961	JAUDPY010000419.1
Botrytis cinerea isolate I9 I9_SCAF__324	74.3	74.3	0.52	3E-12	26.99	2023	JAUDPX010000324.1
Botrytis cinerea isolate Y1 Y1_SCAF__303	74.3	74.3	0.52	3E-12	26.99	2027	JAUDPZ010000303.1
Botrytis cinerea strain Vv3 G31189	74.3	74.3	0.52	3E-12	27.3	2110	JACVGL010001189.1
Botrytis cinerea strain Sl12 T121781	73.9	73.9	0.59	4E-12	27.49	2066	JACVFK010001781.1
Botrytis cinerea strain Sl11 T111712	73.9	73.9	0.59	4E-12	28.97	2111	JACVFL010001712.1
Botrytis cinerea strain Sl9 T9524	73.9	73.9	0.59	4E-12	28.97	2123	JACVFN010000524.1
Botrytis cinerea strain Sl2 T21500	73.9	73.9	0.59	4E-12	27.49	1994	JACVFT010001500.1
Botrytis cinerea strain Sl5 T51774	73.9	73.9	0.59	4E-12	27.49	1998	JACVFR010001774.1
Botrytis cinerea strain Sl8 T81741	73.9	73.9	0.52	4E-12	27.3	1994	JACVFO010001741.1
Botrytis cinerea strain Vv7 G71912	73.6	73.6	0.52	4E-12	27.3	1974	JACVGE010001912.1
Botrytis cinerea isolate B5 B5_SCAF__377	73.6	73.6	0.52	5E-12	27.3	1962	JAUDPV010000377.1
Botrytis cinerea strain BcPgIs-3 scaffold_250	73.6	73.6	0.52	5E-12	27.64	2123	JBGCUU010000250.1

Description	Max Score	Total Score	Query Cover	E value	Per. ident	Acc. Len	Accession
Botrytis cinerea strain Sl3 T31119	73.6	73.6	0.52	5E-12	29.06	2181	JACVGK010001119.1
Botrytis cinerea strain Vv15 G151761	73.2	73.2	0.52	6E-12	27.3	1698	JACVFW010001761.1
Botrytis cinerea strain BcPgIs contig_506	73.6	147	0.52	6E-12	27.64	4099	JARGYR010000506.1
Botrytis cinerea BcDW1 contig_332	73.2	73.2	0.52	7E-12	27.3	1978	AORW01000332.1
Botrytis cinerea strain Hm1 H11516	73.2	73.2	0.52	7E-12	27.3	2082	JACVFH010001516.1
Botrytis cinerea strain Vv10 G101888	72.8	72.8	0.52	1E-11	27.3	1976	JACVGB010001888.1
Botrytis cinerea strain Vv14 G141989	72.8	72.8	0.52	1E-11	27.3	1976	JACVFX010001989.1
Botrytis cinerea strain Vv4 G41718	72.8	72.8	0.52	1E-11	27.3	2055	JACVGH010001718.1
Botrytis cinerea isolate KC25 NODE_938_length_2022_cov_138.591256	72.4	72.4	0.52	1E-11	27.3	2022	JBBMGB010000937.1
Botrytis cinerea strain Vv5 G51615	72.4	72.4	0.52	1E-11	26.38	2145	JACVGG010001615.1
Botrytis cinerea strain Vv12 G121830	72	72	0.52	1E-11	25.46	2038	JACVFZ010001830.1
Botrytis cinerea strain Vv1 G1_22129	70.9	70.9	0.47	2E-11	29.53	1293	JACVGJ010002129.1
Botrytis cinerea isolate KC25 NODE_1089_length_1119_cov_2.453008	69.7	69.7	0.44	3E-11	31.3	1119	JBBMGB010001088.1
Botrytis cinerea strain Rf2 R211694	65.9	65.9	0.24	4E-10	34.11	879	JACVFI010011694.1
Botrytis cinerea isolate R23 R23_SCAF__428	65.1	65.1	0.36	2E-09	29.08	1717	JAUDPY010000428.1
Botrytis cinerea isolate NC7 NODE_993_length_1768_cov_202.015178	65.1	65.1	0.36	2E-09	29.08	1768	JBBMGA010000992.1
Botrytis cinerea isolate BU9 BU9_SCAF__514	65.1	65.1	0.36	2E-09	29.08	1869	JAUDPW010000514.1
Botrytis cinerea isolate Y1 Y1_SCAF__305	65.1	65.1	0.36	2E-09	29.08	1957	JAUDPZ010000305.1
Botrytis cinerea isolate I9 I9_SCAF__328	63.9	63.9	0.36	5E-09	29.08	1929	JAUDPX010000328.1
Botrytis cinerea strain Vv16 G162226	55.1	55.1	0.41	0.000002	25.93	1161	JACVFV010002226.1
